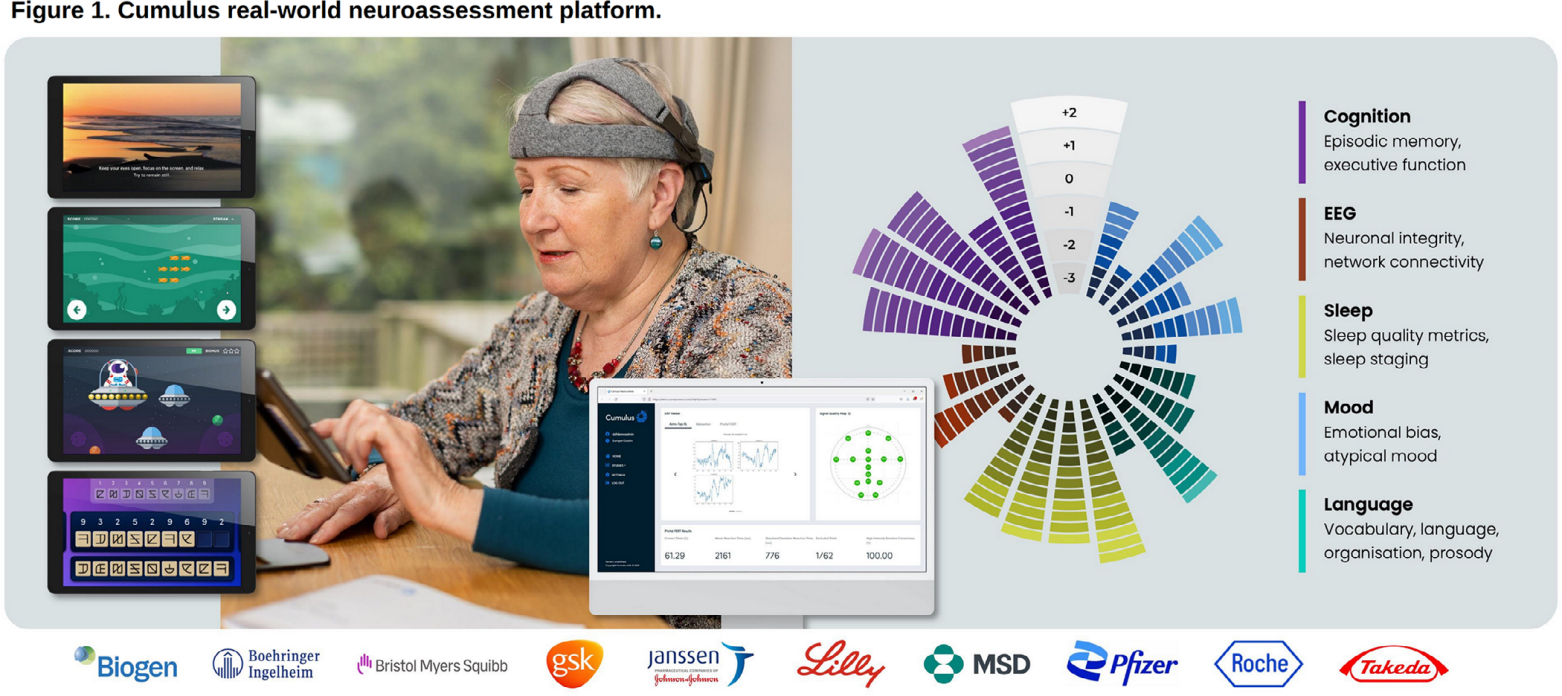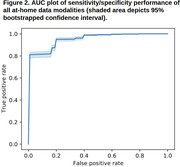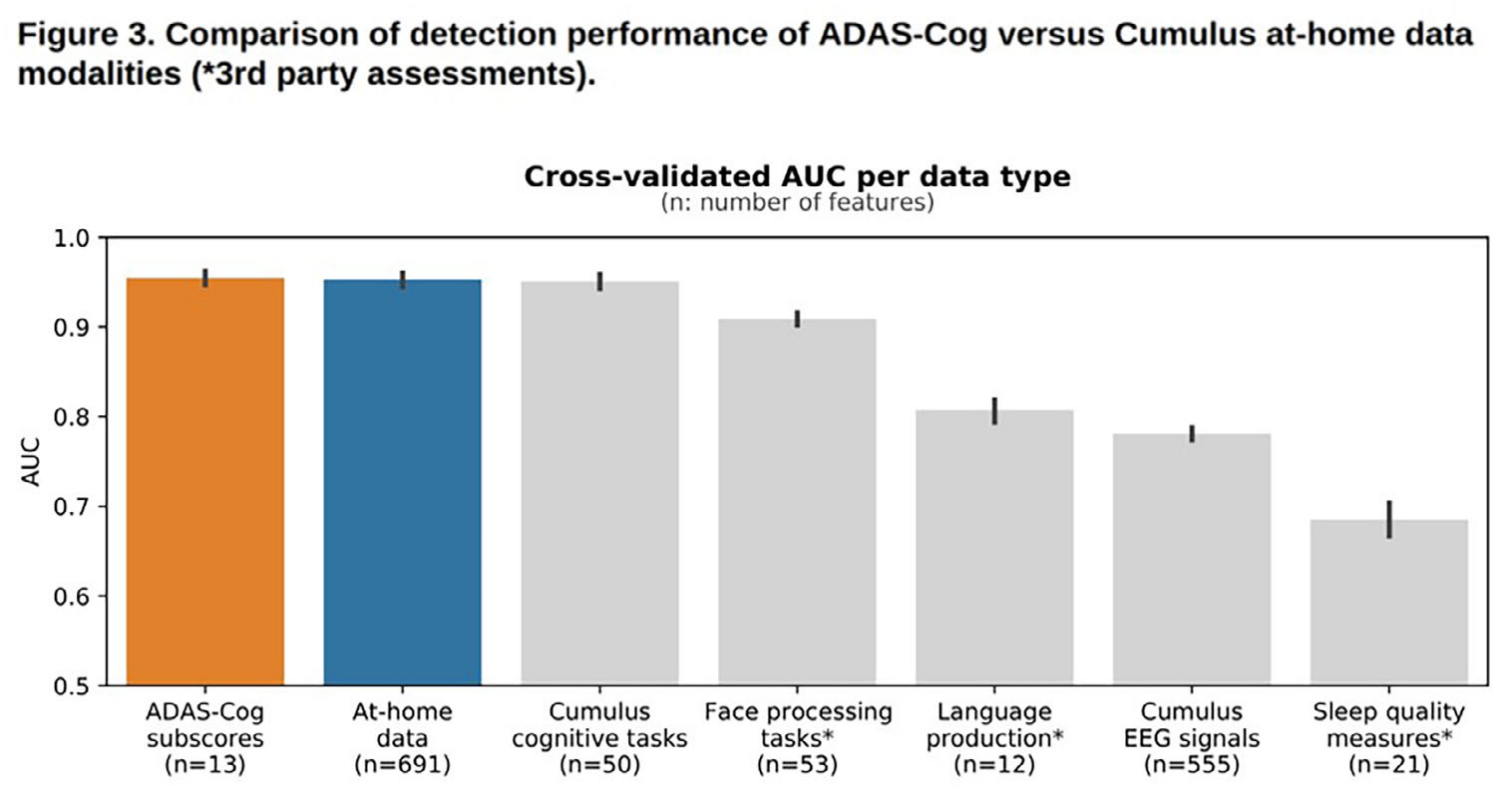# Machine‐learning analysis of real‐world multimodal data collected autonomously at home detects dementia as precisely as a traditional composite scale

**DOI:** 10.1002/alz.092145

**Published:** 2025-01-09

**Authors:** Laura M Rueda‐Delgado, Alison R Buick, Hugh Nolan, James B Rowe, Brian Murphy

**Affiliations:** ^1^ Cumulus Neuroscience, Dublin Ireland; ^2^ Cumulus Neuroscience, Belfast UK; ^3^ Department of Clinical Neurosciences, University of Cambridge, Cambridge UK

## Abstract

**Background:**

Current tools for Alzheimer's disease screening and staging used in clinical research (e.g. ACE‐3, ADAS‐Cog) require substantial face‐to‐face time with trained professionals, and may be affected by subjectivity, "white coat syndrome" and other biases. Alzheimer's symptomology is multi‐factorial and varies day‐to‐day. To enable stratification for precision treatments, more effective composite endpoints are required, with accurate quantification of functional impairment in individual domains. Ideally these would be measured objectively and frequently in real‐world contexts to improve validity, and to reduce clinical burden in trial and care settings.

**Method:**

We present a machine‐learning stratification analysis using data from the Cumulus real‐world multi‐domain neuroassessment platform, in CNS101, a year‐long study designed with a consortium of 10 pharma companies. Participants completed a range of tablet‐based functional tasks with wake EEG, and separately recorded EEG during sleep. This cross‐sectional analysis uses 1111 sessions from repeated sampling with 101 participants (47 patients with mild Alzheimer’s type dementia, and 54 corresponding controls, recruited across 7 sites in the UK), during an early two‐week burst period of autonomous at‐home use. Bagging of decision trees was used to compare the power of different data sources in discriminating dementia from neurotypical states. This algorithm is widely used for its performance across learning tasks (linear and non‐linear), and heterogeneous datasets of differing size, levels of noise, and collinearity. For each set of input data, 100 classifiers were trained and evaluated (10‐fold cross‐validation in each of 10 random partitions), and the mean Area Under the Curve (AUC) of sensitivity/specificity trade‐off was calculated.

**Result:**

A full set of 691 multimodal features yielded a classifier with AUC performance of 0.953 and error rate 4.7% – compared to AUC of 0.955 and error rate 4.5% for the set of 13 ADAS‐Cog subscores. While individual modalities/domains all performed above chance, the 50 features from Cumulus cognitive tasks alone had an AUC performance of 0.951.

**Conclusion:**

Patients using digital technology autonomously in the home can yield data that matches the discriminative power of a traditional clinician‐administered composite scale. This can enable objective precision measurement of disease severity at scale